# Forest Fire Smoke Detection Based on Deep Learning Approaches and Unmanned Aerial Vehicle Images

**DOI:** 10.3390/s23125702

**Published:** 2023-06-19

**Authors:** Soon-Young Kim, Azamjon Muminov

**Affiliations:** 1 Department of Physical Education, Gachon University, Seongnam 13120, Republic of Korea; klpga0166@gachon.ac.kr; 2 Department of Computer Engineering, Gachon University, Seongnam 13120, Republic of Korea

**Keywords:** forest fire, smoke detection, wildfire smoke, deep learning, remote sensing, CBAM, decoupled head, UAV images, YOLOv7

## Abstract

Wildfire poses a significant threat and is considered a severe natural disaster, which endangers forest resources, wildlife, and human livelihoods. In recent times, there has been an increase in the number of wildfire incidents, and both human involvement with nature and the impacts of global warming play major roles in this. The rapid identification of fire starting from early smoke can be crucial in combating this issue, as it allows firefighters to respond quickly to the fire and prevent it from spreading. As a result, we proposed a refined version of the YOLOv7 model for detecting smoke from forest fires. To begin, we compiled a collection of 6500 UAV pictures of smoke from forest fires. To further enhance YOLOv7’s feature extraction capabilities, we incorporated the CBAM attention mechanism. Then, we added an SPPF+ layer to the network’s backbone to better concentrate smaller wildfire smoke regions. Finally, decoupled heads were introduced into the YOLOv7 model to extract useful information from an array of data. A BiFPN was used to accelerate multi-scale feature fusion and acquire more specific features. Learning weights were introduced in the BiFPN so that the network can prioritize the most significantly affecting characteristic mapping of the result characteristics. The testing findings on our forest fire smoke dataset revealed that the proposed approach successfully detected forest fire smoke with an AP_50_ of 86.4%, 3.9% higher than previous single- and multiple-stage object detectors.

## 1. Introduction

The forestry industry is a critical feature of the global system, being an essential resource for both human well-being and progress, as it plays a crucial role in ecosystem functioning. Consequently, protecting forest resources is therefore necessary for human existence and development and for preserving Earth’s ecosystems in a constant balance. Forest fires pose a significant threat as they spread rapidly and are challenging to extinguish due to the large amount of combustible material. These fires cause severe damage to human life and property and accelerate the degeneration of the ecological environment [1]. Therefore, the early detection of forest fires is crucial for reducing disasters. Image-based fire detection methods are particularly suitable for outdoor environments, such as forests, mountains, and parking areas, in contrast to sensor-based approaches [2]. These techniques are often classified as either smoke detection or fire detection [3]. The latter is essential for early fire detection as smoke spreads faster than a flame and covers a more extensive area [4]. Conventional image-based fire smoke detection methods rely on low-level handcrafted features, such as color, texture, shape, and others based on experience. However, these scenario-specific methods exhibit a decreased accuracy rate when environmental conditions change [5].

Summer is the season that is the most commonly associated with forest fires due to drought, low plant water content, and increased human activity in forested areas. However, significant and intermediate mountain regions are particularly vulnerable to forest fires in the late fall, winter, and early spring. A fire requires three things in proper proportions to start and spread: fuel, which can be any combustible object; a source of heat, such as a flame or spark; and oxygen to feed the fire. Devastating everything in their path, uncontrolled wildfires may travel great distances, even crossing rivers and roadways [6]. Wildfires annually damage between four and twelve million miles or sixty-five to eighty thousand acres. The intensity of forest fires determines the scope of their environmental damage, and the causes of forest fires can range widely [7].

When forest fires are not put out immediately, the resulting destruction and cost of fighting them rise dramatically as a result [8]. To lessen the severity of wildfires, the time it takes to report a blaze is of paramount importance [9]. Official agencies receive help from a wide range of terrestrial and spatial technology to detect wildfires at an early stage and identify their exact position [10]. Nevertheless, these systems have limitations that may reduce their fire detection efficacy. Therefore, to lessen the devastation of forests and their resources, we need to design new methods for observing forest fires and enhance our fire management tactics. The fast development of computer vision technologies and artificial intelligence (AI) has led to considerable advances in deep learning for various complex visual tasks [11,12,13,14]. Image classification [15], object identification [16], semantic segmentation [17], and other applications [18] are only some of the computer vision problems that have benefited from the use of machine learning. Recently, the convolutional neural network (CNN) based smoke detection algorithms have received much attention and have made significant improvements in the accuracy with which they identify smoke from fires.

There are several ways in which fires can exhibit a significant effect on biodiversity. They release much carbon into the atmosphere, raising temperatures and altering animal and plant life [19]. Fires can negatively impact ocean infrastructure, coral reefs, and other underwater environments due to the way in which they can change biomass accumulation in ecosystems and disturb the natural cycle of water [20]. Also detrimental to the well-being of humans and animals is the fact that smoke from forest fires may greatly limit the production of photosynthesis [21]. The Amazon jungle, for instance, is home to an astonishing number of both plant and animal species, many of which have not been thoroughly described. Repeated forest fires therefore constitute a significant danger to the unique species that exist there. All of these effects make combating fires more difficult, which is a significant problem [22].

Compared to the traditional methods of detecting smoke in images for forest fire detection, modern approaches using CNNs can extract depth features automatically, thereby making them more adaptable to variable wilderness environments. However, these methods can have a higher false alarm rate when the amount of smoke in the image is small. This is because CNNs can have difficulty focusing on small smoke and may be more likely to detect the background of the image rather than the smoke itself. Additionally, images in these environments often contain scattered areas, such as shadows, clouds, haze, and fog, which can be challenging for small smoke detection.

Unmanned aerial vehicles (UAVs) versatility, speed, and precision in spotting forest fires have led to their widespread adoption. With their ability to fly at low altitudes, UAVs can capture high-resolution images of forest areas, thereby making it possible to identify fires early. UAVs also have the ability to fly in dangerous, inaccessible regions [23]. They are able to carry various cameras and sensors that can detect the different wavelengths of light, including infrared, which can detect heat sources that are not visible to the naked eye. In addition, UAVs can be equipped with real-time communication systems that allow firefighters to respond quickly to fires and provide them with helpful information regarding the fire’s location, size, and movement [24,25]. Overall, using UAVs in forest fire detection is becoming increasingly important, and is likely to play an even more significant role in the future of wildfire management.

In addition to their widespread use in forest fire detection, deep learning systems have shown better performance than classical image processing techniques across a number of other contexts. To serve as a classifier for finding forest fires, Pan et al. [26] developed an AddNet [27]. To determine the scope and location of the blaze, they segmented photos into many tiny patches and categorized them with a great precision. Zhang et al. [28] used a cascade strategy and a combined classifier to detect whole pictures and fine-grained patches. The effectiveness of YOLOv3 [29] for forest fire detection was enhanced by Valikhujaev et al. [30], and it has since been applied to UAVs for forestry surveillance. Wu et al. [31] made an analogous inquiry into the efficiency of several detectors involving YOLO, SSD [32], and Faster R-CNN [33]. They modified the YOLO network’s architecture to speed up and more accurately detect fires.

Xu et al. [34] used an ensemble learning method to improve the precision of detecting forest fires while reducing the frequency of false positives. However, this approach was very complicated and needed to respond more adequately. Other methods presented here have effectively located and identified areas where fires have occurred in photos. Additionally, several studies have used semantic segmentation techniques to provide pixel-level information on fire zones, which provides more extensive and accurate coverage. The ATT Squeeze U-Net, suggested by Zhang [35], is an attention-based variant of the original U-Net that aims to enhance accuracy without sacrificing the network’s compact size. Real-time speed, efficiency, and high accuracy were all found to have been attained when the compressed fire binary categorization model proposed by Song [36] was implemented in an embedded device.

Identifying wildfires using UAV photos requires developing a deep-level network approach for obtaining abstract data from pictures. However, training a deep neural network can be tough and time intensive when just a small amount of labelled data is available. Transfer learning, which entails applying a pre-trained model and modifying its parameters to suit the new job, is the answer to this problem. Since labeled data is in short supply, transfer learning can solve the overfitting issue. Through utilizing the information learned from pre-trained models, transfer learning may considerably speed the training process and thereby improve the model’s performance on the new task. Therefore, transfer learning is an effective strategy for training deep learning models for image analysis tasks, and especially requires more labeled data.

To overcome these limitations, this research proposes a new method for identifying forest fire smoke using photos captured by UAVs. Our system uses the modernized YOLOv7 model to detect smoke from forest fires. We used pre-trained weights as initialization parameters for the backbone network and modified the network structure parameters to boost the performance of the classic YOLOv7 model. Deploying the improved network to a forest fire smoke dataset allowed us to identify hazardous chemicals, such as smoke reliably. Section 3 and Section 4 detail how we improved the YOLO7 model and ran tests on an AI server.

The following are the significant findings from this research:

- To enhance the performance of the smoke detection model, a massive collection of forest fire smoke photos was collected using pictures taken from UAVs and forest land pictures;
- A completely automated forest fire smoke detection model was built using deep learning algorithms and YOLOv7 to decrease natural disasters and wildland resource loss;
- We adopted the CBAM module after evaluating the effects of three different attention mechanisms on the model’s output. The decoupled head and CBAM attention mechanism were verified to be successful;
- We applied SPPF+ and BiFPN modules to concentrate on small-size forest fire smoke and distribute the feature information more evenly across the scales. Improved sensitivity to localized smoke is one of the many benefits of the BiFPN feature fusion approach.

This paper is organized as follows: Section 2 examines the current research on UAVs and deep-learning smoke detection strategies for forest fires. In Section 3, we describe the experimental dataset we employed and break down the inner workings of the YOLOv7 model in greater depth. In Section 4, we explore the experimental results in depth and compare the efficiency of our proposed system to that of other current smoke detection systems. Lastly, we present a discussion and brief overview of the work in Section 5 and Section 6.

## 2. Related Works

The conventional point sensors utilized in early fire smoke detection systems are inadequate for effectively detecting smoke signals in larger spaces. In the case of a forest fire, substantial plumes of smoke are emitted into the environment, making a properly operating smoke warning essential for preventing casualties. Climate change exacerbates the risk of catastrophic wildfires, which may have far-reaching effects on human populations, ecosystems, and the economy if they are not extinguished quickly. Two methods are available for monitoring wildfires: detecting smoke and flames. Smoke serves as the primary indicator of an impending wildfire, necessitating early warning and detection systems that are sensitive to the smoke in the operating environment, such as deep-learning models. Despite this, advancements in technology have enabled the development of modern methods for detecting smoke and fires, taking into account the availability of processed data, as previously reported by several studies.

Previous research [37] have stated that the advent of deep learning techniques makes it possible to detect forest fires using image analysis, thereby providing novel insights for wildfire alert systems. It has been shown that deep learning techniques can detect shifts in smoke photos by extracting features from labeled photographs [38]. In [39], the authors proposed a method for detecting wildfires by utilizing UAVs to collect imagery of the area, which would subsequently be evaluated with the YOLOv3 model and a modest CNN. As the trial findings revealed, the UAV system and the deep learning-based fire detection technique exceeded their expectations regarding accuracy and speed, with the test results for the algorithm’s accuracy in recognition being close to 83%.

YOLOv5 was updated in [40] to incorporate adaptive anchor training through the K-means++ technique to increase the detection speed and performance and reduce fire damage. The authors subjected three different YOLOv5 models—a small one, a medium one, and a large one—to various loss functions, namely CIoU and GIoU, respectively. Initially, they used a synthetic approach to grow their collection of 4815 images to include 20,000 images. The enhanced model performed 4.4% better on average than the baseline YOLOv5, with the CIoU loss function producing the highest results (mean accuracy of 86.0% and recall of 78.0%, respectively).

The study in [41] also offered a method for detecting fires at all hours of the day and night using an improved YOLOv3 model, but with a larger detection area and a faster detection speed. This study emphasized the need to gather accurate information to determine fires correctly, and it achieved it by amassing a dataset of 9200 images from a wide variety of places, such as Google image archives and publicly available sources, as well as stills from videos. The database’s overall size was increased using data augmentation methods, including image rotation. This technique relied on a custom set of cameras and the YOLOv3 model for real-time detection of fires, with a mean precision of 98.9%. However, it is still a challenge for researchers to identify non-fire flame characteristics, such as the presence of bright light or high-beam lamp illumination.

A novel ensemble learning framework for detecting forest fires was introduced in the study [42]. Publicly available data was used as the basis for the framework’s significant learners, Yolov5, and EfficientDet, with EfficientNet having been used for both detection and classification. The authors collected 10,581 photos from widely used resources, such as VisiFire and FD-dataset to create their dataset. The research showed that, compared to other models, its fire detection accuracy was significantly higher, with an average precision of 79.7% at an IoU of 50%. Nevertheless, the suggested approach was flawed in incorrectly classifying the sun as a fire during sunset. The authors then proposed a new framework, Swin-YOLOv5 [43], that uses a transformer over three heads to improve the extraction of features in the original YOLOv5 design, and thereby address the limits of the previous [42] model. The research compared seven hyperparameters using a dataset of 16,503 images from two target classes. Swin-YOLOv5 beat the baseline model statistically, with an average precision increase of 4.5% points and an average accuracy increase of 0.7% for IoUs between 0.5 and 0.95, respectively. Together, these results show that ensemble learning approaches have the potential to enhance the accuracy of forest fire detection. However, further research is necessary to address the limitations of these models and explore alternative approaches for enhancing the performance of these systems.

Detection frameworks have been the subject of further research into developing specific methods for interior and exterior item detection and classification that may be applied to detect flames and smoke. One such study is [44], which presented a unique architecture for identifying items within occupied spaces. By utilizing the anchor-free technique for variable minimization and the VariFocal loss for data weighing, this suggested design made optimal use of the YOLOv5 model. In addition to the publicly available dataset, the authors offered a newly built sample with 11,367 images divided into training, testing, and validation sets, respectively. The popular Pascal-VOC2012 dataset was used throughout the study. Decoupling the head’s layer was one component of the YOLOv5 improvement that was implemented to enhance the detection accuracy and efficiency, which was accomplished using a size of 640 × 640 pixels. Eleven existing models that used YOLO across multiple formats were then compared to the findings of the new system. The evaluation results showed that the suggested system successfully identified internally occupied items with an average precision of 93.9% at an IoU of 0.9. This research provides evidence that altering the model design and training data of the YOLOv5-based system can improve its ability to identify flames and smoke.

In the research presented in [45], a real-time experiment was conducted to detect internal and external objects by designing a system that employs camera sensors commonly used in Lidar devices, such as OS1-64 and OS0-128. The primary innovation was high-resolution (2048 × 128), 360 degree panoramic photos. Results from the created system were compared between Faster R-CNN, Mask R-CNN, YOLOx, and YOLOv5, respectively. Individuals, bicycles, seats, and automobiles were isolated from the sensor images for indoor and outdoor usage. When compared to other models, YOLOx performed the best in terms of accuracy (over 81%), precision (over 99%), and recall (over 95%) while recognizing indoor and outdoor items. According to the study, YOLOx is faster and more accurate than YOLOv5.

The authors in [46] devised an ABi-LSTM model to detect forest fire smoke. This model comprises a spatial features extraction network, a bidirectional extended short-term memory network, and a temporal attention subnet. The network for extracting spatial characteristics from candidate patches use the ViBe method for removing unnecessary background information. Long short-term memory (LSTM) networks similar to this use spatial data to learn about smoke over time, whereas attention networks focus on finding discriminative images. When applied to 1920 × 1080 videos from a forest fire monitoring system, the Abi-LSTM model produced a 97.8% accuracy rate, outperforming the image-based deep learning model by 4.4%.

According to the study [47], there needs to be more adequate and high-quality data in research archives for detecting fires and smoke. The absence of development- and use-ready labeled data significantly contributes to this shortcoming. NEMO (Nevada Smoke Detection Benchmark) is the first data repository of its sort and was offered as an innovative solution to this issue. NEMO uses a database of aerial photographs collected from the detection sites to determine wildfires. The dataset includes 7023 images taken by different cameras at different times and places and were used for fire detection. The data was evaluated using many different detection models. These included Faster R-CNN and RetinaNet. The results showed a sensitivity of 98.4% within 5 min and a specificity of 42.3% on average for detection, respectively. Notably, NEMO was designed to work with photos of varied sizes, including those that are horizontal, far away, or in-between.

The study’s authors [48] considered six different CNN architectures when creating a wildfire inspection system that could estimate geolocation using a cheap commercial UAV. These architectures were Inception v3, VGG16, MobileNet v2, DenseNet, and ResNet 50. There are three primary phases to the planned structure, beginning with a tablet’s graphical user interface (GUI), with which the operator initially defines the search area. Once the drone has the location’s coordinates, it may fly there and inspect to collect further information. Using this information, the quadcopter pinpoints the location of the fire and sends back its coordinates along with a live video feed of the search area to the operator’s tablet computer. Based on the assessment, the system’s accuracy was 99.29% for the Inception v3, 99.38% for the ResNet-50, 99.74% for the VGG-16, 99.47% for the MobileNet v2, 98.9% for the NASNetMobile, and 99.65% for the DenseNet, respectively.

In order to identify forest fires using UAVs, Ali Khan et al. suggested a dataset and benchmark which they named as DeepFire [49]. When a fire is detected, the UAVs will coordinate with other UAVs in the area and send the information to a far-off wildfire catastrophe administration center. There are a total of 1900 images in the DeepFire dataset, 950 of which are classified as fire and 950 as non-fire, respectively. The authors used transfer learning with the VGG19 architecture to improve the prediction precision. Simulation data showed that the suggested method was highly effective, with a precision of 95.7%, an accuracy of 95%, and a recall of 94.2%, respectively.

Diyana et al. [50] established a forest monitoring system to aid in early fire detection and assessment. UAVs with optical, thermal, or both types of cameras are used for this platform, which can be either fixed- or rotary-winged. To confirm possible fire spots, rotary-wing drones operate at a lower height, while fixed-wing drones fly a medium-altitude assessment of the monitored region. The detection of a fire causes an alarm to go off, which notifies the appropriate authorities on the ground. With a model built on the SSD, employing MobileNetv1 as its backbone, and the COCO dataset as weights, the authors reached a 94% accuracy.

Panagiotis et al. [51] introduced a method for fire detection that uses a 360 degree aerial digital camera placed on a UAV to capture footage from an infinite swath of sky. Images taken by the optical camera are projected onto an isosceles rectangle before being converted to a stereographic form. Two different DeepLab V3+ architectures were then used to conduct research on flame and smoke segmentation. Consideration of the image’s aesthetics was used to verify the identified areas. The authors created a dataset for 360 degree fire detection using 150 equirectangular images. The experiment showed that the suggested system had a 94.6% F-score.

Alireza et al. [52] curated the FLAME dataset, which consists of fire-related visual data in Northern Arizona taken by drones. This database employs regular and thermal cameras and captures aerial videos and images in four color palettes: fusion, regular, green-hot, and white-hot. The authors developed a CNN approach, which yielded a classification accuracy of 76% for frame-based fire identification. Additionally, segmentation techniques were used to accurately detect fire borders, resulting in a precision of 92% and a recall of approximately 84% for the FLAME system, respectively.

Several studies have explored the potential of satellite-based object detection methods, which can be used alongside video cameras and drones to improve the detection of wildfires across large areas. The framework [53] was created to find safe landing areas for the UAVs and examined the usefulness of several versions of YOLO in finding acceptable landing areas. The DOTA database was utilized, which includes 11,270 satellite images with high-resolution and 15 labels. According to the findings that were obtained, YOLOv5 with large network weights outperformed the competition with an accuracy rate of 70%, a recall rate of 61%, and a mean average accuracy rate of 63%, respectively.

Yifan et al. [54] introduced Light-YOLOv4, a lightweight real-time flame and smoke detector. First, they used a more lightweight backbone network than YOLOv4. Second, they added bidirectional cross-scale connections. Finally, they partitioned the convolution and independently computed the channel and geographical region, all of which are improvements over YOLOv4. Light-YOLOv4 had an accuracy of 86.43% when detecting flames and 84.86% when detecting smoke, respectively. Furthermore, it also had a mean average precision of 85.64% at a confidence threshold of 0.5 for flame and smoke detection, and a mean average precision of 70.88% when detecting smoke at the same confidence level, respectively.

## 3. Materials and Methods

### 3.1. Dataset Acquisition for Forest Fire Smoke Detection

The proper dataset preparation plays a vital role in successfully implementing the algorithm, as presented in this paper. It is worth noting that deep learning model accuracy heavily relies on the quality of the images utilized in the training and testing processes. Our evaluation of forest fire smoke images revealed that vision-based systems had inadequacies in their datasets, and existing open-access datasets also had issues. To ensure that our learners were capable of detecting various sizes of forest fire smoke, we utilized forest fire smoke images [18,55], wildland images [56] for non-wildfire photos, and other web-based images. These datasets were obtained through crawling pictures or videos captured by a UAV, as the forest fire smoke model was developed to utilize the UAVs for monitoring purposes.

**Figure 1:**
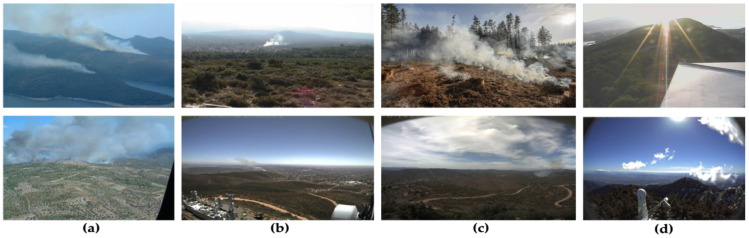
A sample of images from the forest fire smoke dataset. (**a**) Large and medium smoke; (**b**) small smoke where the attention of smoke is high in the center and low at the edge; (**c**) low smoke density makes it hard to make out details, including color, edge, and texture; and (**d**) non-smoke images captured under different weather conditions including sunny and cloudy.

The images collected for this study primarily consisted of aerial photos of wildfire smoke and forest backgrounds. The size of pictures varied between 2048 × 3072 and 512 × 512 pixels, respectively. The images depicted recent wildfires in the world. This diverse dataset made the algorithm more generalizable in complex forest environments. After undergoing manual filtration, we created a single integrated dataset consisting of 3500 forest fire smoke pictures and 3000 non-forest fire smoke pictures, respectively. All images were then resized to the size of 640 × 640 pixels. These findings are depicted in Table 1, and Figure 1 demonstrates the sample images of the forest fire smoke dataset which display variability in the shape and size of smoke in natural environments, which can lead to misclassification by the conventional detection methods.

Large- and medium-sized forest fire smoke images are shown in Figure 1a. In contrast, Figure 1b displays images with a small smoke size, a high concentration in the smoke center, and a low concentration at the edge, making the determination of the extent of the smoke challenging. Figure 1c shows an example of a picture with a low concentration of smoke, where the edge, texture, color, and other properties of smoke are not very noticeable. Finally, Figure 1d shows non-smoke images captured under different weather conditions, such as sunny and cloudy conditions. In conclusion, standard smoke detection systems need help to effectively identify smoke due to the variety in form and quantity of smoke in natural surroundings. Therefore, it is essential to create a smoke detection algorithm for forest fires that works well with photos of smoke that exhibit in various forms due to its origin in the natural world.

A substantial amount of labeled training data is crucial to the success of a deep learning model. However, it might be difficult to achieve trustworthy findings for wildfire smoke detection with such datasets due to overfitting, class imbalance, or inadequate data. Failure to capture visual patterns reliably by a model is known as overfitting. Image data augmentation, in which existing photos are tweaked and reused to boost a model’s accuracy, was used to solve this problem. A survey of the relevant literature [57,58] revealed that geometric changes, including flipping and rotation, are the most valuable methods for improving picture data. The number of photos in the forest fire smoke detection dataset was increased by experimentation and applying data augmentations such as rotation and horizontal flips [59,60]. The CNN’s model performance is very sensitive to the amount and quality of the picture datasets used to train the models.

We added multiple adjustments to each original fire image to improve the model’s generalization of the preceding training photos and allow it to learn from a broader range of events. These adjustments included horizontal flip and counterclockwise rotations of 60 and 120 degrees. Furthermore, we incorporated training photos portraying non-smoke but comparable scenes, such as mountains, clouds, fog, and others, in an effort to reduce the frequency of false positives.

For our objectives, we employed a dataset of 6500 photos to identify forest fire smoke, splitting it into a training set of 5200 images and a test set of 1300 images, respectively. Data augmentation techniques were used exclusively on the training set to enhance its size. According to Table 2, this meant that there were a total of 32,500 photos available for use in identifying smoke from forest fire.

### 3.2. Overall Architecture of Forest Fire Smoke Detection

**Figure 2:**
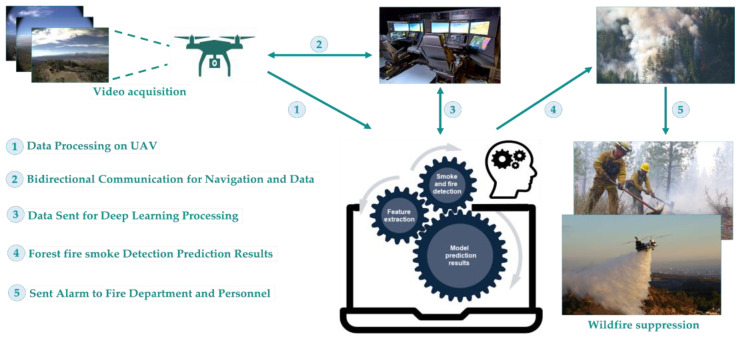
Framework of the forest fire smoke detection.

Deep learning’s effectiveness in identifying forest fire smoke has been proven compared to the more conventional techniques. However, further research into the many characteristics of smoke is required in order to enhance the detection accuracy in dense wildland scenes. In this study, unlike previous research, our focus is on extracting more detailed smoke features to distinguish smoke from complex backgrounds, such as clouds and fog. The primary goal of this research was to implement the most efficient detection models for smoke detection, with the capability to notice smoke from different distances, including close, medium, and distant areas. As shown in Figure 2, the suggested framework incorporates a flowchart of the approach developed to decrease the sensitivity of the detection methods employing prospective data by integrating data augmentation strategies. We also set aside 20% of the dataset specifically for testing and assessing detection outcomes, which should thereby help keep the detection more consistent. Due to its comprehensive nature, the suggested approach could be used with a variety of different datasets.

In order to determine the most effective and precise model for a specific task, comparing detection models is therefore crucial. This is especially crucial in detecting smoke in forest fires, where the precision of detection models can have significant impacts. This study aimed to develop detection methods for forest smoke and compare them with single- and multiple-stage object detectors, such as YOLOv5, DeepSmoke, Mask R-CNN, and Cascade R-CNN using our smoke images. By employing the same dataset, the researchers guaranteed that any differences in the model’s performance are attributable to the model’s architecture and not the dataset. This methodology could also be applied to other applications, such as monitoring warning signals during fire outbreaks using detection models coupled with UAVs and detecting fires early via smoke detection. Overall, comparing detection models is crucial for developing accurate and efficient models for specific applications, and has significant real-world implications.

In order to increase the accuracy of early forest fire smoke detection in various weather conditions, including foggy, hazy, and sunny, the usage of UAVs equipped with cameras for collecting images and videos have been integrated with deep learning and computer vision algorithms. We proposed forest fire smoke detection using UAV images and built an optimized YOLOv7 model. Images captured by the UAVs are transmitted to a base command post. They are then processed by an AI system equipped with deep CNNs to identify the existence of fire or smoke. This system provides a high accuracy in detecting smoke regions and executes real-time image processing quickly due to its powerful processor. The steps involved in utilizing the UAV camera and computer to identify smoke from forest fires are shown in Figure 1. Deep learning procedures have been used to replace the conventional methods, thereby simplifying feature extraction and detection considerably.

Once the image has been acquired and preprocessed, the next step was to extract the pixels that corresponded to the object of interest, such as smoke and fire. Feature extraction involves identifying image characteristics, including motion, colors, corners, edges, brightness levels, and intensities, which are all relevant to the object of interest. This method allows for a more in-depth inspection of the segmented image to pinpoint the relevant details. A trained AI model was then used to analyze the input image for patterns that indicate the existence or absence of smoke. If the system returns a smoke existence result, the system then sends an alert to the fire department through the UAVs or base command post. Overall, feature extraction and pattern recognition using deep learning models play a crucial role in early wildfire detection and notification systems.

### 3.3. Original YOLOv7

**Figure 3:**
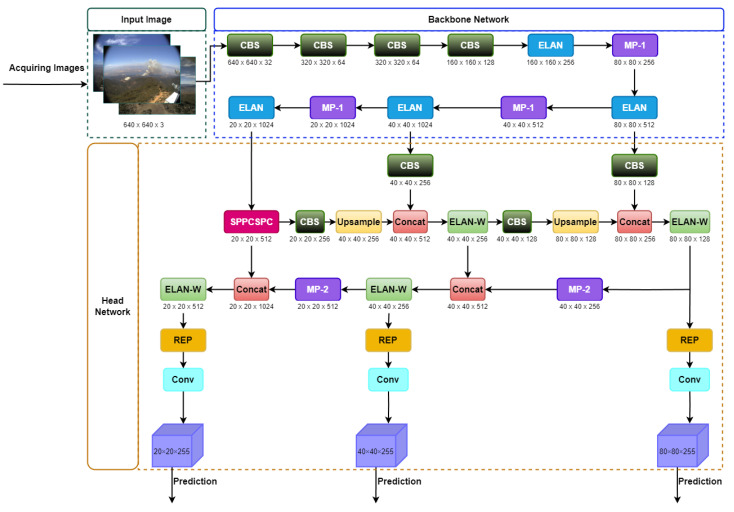
Network architecture of the YOLOv7 object detector.

YOLOv7 is considered an advanced object detector due to its remarkable performance on publicly available datasets [61]. Figure 3 depicts the two primary parts that make up the YOLOv7 model: the backbone network and the head network. The original picture undergoes preprocessing before being sent into the backbone network. The input pictures are evenly scaled to a 640 × 640 size during the preprocessing stage using hybrid and mosaic data augmentation methods and the adaptive anchor frame estimation approach introduced by the YOLOv5 model. This ensures that the input size satisfies the criteria of the backbone network. Features are extracted using the backbone network and then fused in the head network.

**Figure 4:**
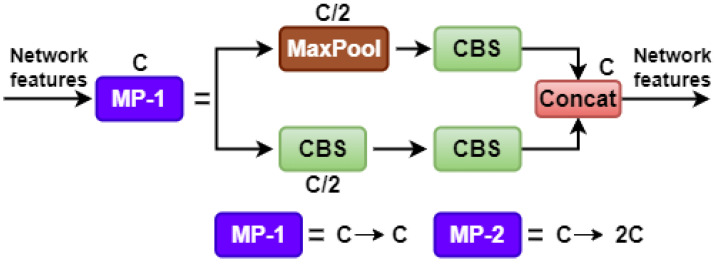
Structure of the MP.

The backbone network in the YOLOv7 model consists of three key components: extended efficient layer aggregation network (E-ELAN), CBS, and MP-1. The CBS module consist of convolution, batch normalization, and SiLU activation function. The E-ELAN component keeps the gradient route from the initial ELAN design and allows the network to learn a wider variety of features through the guidance of various feature group computational blocks. Upper and lower branches make up MP-1, which is made up of CBS and MaxPool, as shown in Figure 4 (where C is the channel). The MP structure is applied for a down-sampling purpose. Both the picture’s width and length are reduced due to the usage of MaxPool, and the number of channels in the image is cut in half according to CBS’s 128 output-channel configuration, which is used by the top branch. The bottom branch employs a CBS algorithm with a 1 × 1 stride and kernel to down-sample the picture channels, a 2 × 2 stride and 3 × 3 kernel to down-sample the picture width and length, and a concatenation operation to merge the features recovered from the two branches. Both MaxPool and CBS improve the network’s feature extraction capacity by extracting the maximum and minimum value information from tiny local regions, respectively.

The feature pyramid network (FPN) architecture and the E-ELAN are used in YOLOv7’s head network to extract and fuse features from various backbone levels. Attributes are extracted at multiple scales using the spatial pyramid pooling (SPP) structure while computation costs are reduced, and feature extraction is enhanced using the convolutional spatial pyramid (CSP) design. By fusing these two methods, we obtain the SPPCSPC, which is utilized to broaden the network’s perception. The feature extraction process receives additional enhancements by the ELAN-W layer. The MP-2 block is analogous to the MP-1 block, except that it contains two extra output channels. The number of image channels utilized in the features generated by the head network is modified using the Rep structure, and a 1 × 1 convolution is used to estimate category, confidence, and anchor frame. Ultimately, network complexity is decreased without losing in predictive performance, as the Rep structure uses a specific residual design inspired by RepVGG [62], which could be simplified to a simple convolution in actual estimations.

### 3.4. Attention Mechanism Module

In deep learning, the attention mechanism has commonly been used for drawing attention to the critical details by discovering hidden patterns in the raw data. This method has seen extensive usage in the field of computer vision, where it has been implemented in the form of multi-order attention, pixel attention, and channel attention, respectively. However, channel-only approaches to attention control, such as squeeze-and-excitation (SE) [63], ignore location information that is essential for visual activities. To address this limitation, researchers developed the convolutional block attention module (CBAM) [64], which builds on SE but incorporates location information through global pooling on the channels.

**Figure 5:**
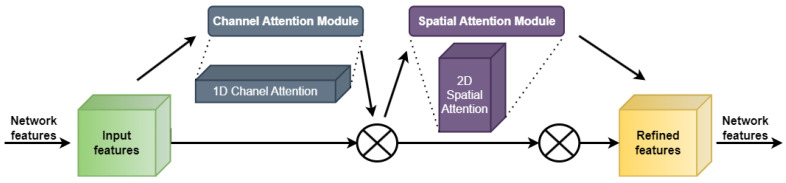
Diagram of the CBAM structure.

The CBAM module is an attention mechanism that can focus on both the spatial and channel dimensions. As shown in Figure 5, it is made up of two distinct parts: the CAM (channel attention module) and the SAM (spatial attention module). The CAM emphasizes the foreground and meaningful regions of the image, while the SAM focuses on the positions that contain contextual information for the entire image.

There are two phases to the CBAM attention mechanism: 1D channel attention (dark grey box) and 2D spatial attention (dark purple box). In the Figure 5, ⨂ denotes element-wise multiplication. During multiplication, the attention weights are accordingly broadcasted (copied): channel attention values are broadcasted along the spatial dimension, and vice versa. Two 1 × 1 C feature maps are produced from the H × W × C input feature map using global max pooling (GMP) and global average pooling (GAP) in the channel attention module. These feature maps are then inputted into a two-layer multilayer perceptron (MLP), with the first layer consisting of C/r neurons (with r representing the reduction rate) and employing the ReLU activation function, and the second layer consisting of C neurons, with the weights of both levels being equally distributed across the network. The output characteristics are accumulated piecemeal, and the final channel attention feature is generated through a sigmoid activation function. The input feature for the spatial attention module is calculated by multiplying the output of the channel attention feature with the input feature map. This procedure is illustrated by Equation (1).

The output of the channel attention module is fed into the spatial attention module of the CBAM to create a feature map. Two feature maps of size H × W × 1 are first generated using global max pooling and global average pooling techniques. The dimensionality of these feature maps is lowered by joining them. The spatial attention feature is then generated using sigmoid activation on the resultant feature map. The output feature map is calculated by multiplying the input feature map by this spatial attention feature. Equation (2) is a model for this operation.

(1) $$M_{c}\left(F\right)=\sigma (MLP\left(AvgPool\left(F\right)\right)+MLP(MaxPool(F)))$$

(2) $$M_{s}\left(F\right)=\sigma (f^{7\times 7}\left(\left[AvgPool\left(F\right);MaxPool\left(f\right)\right]\right))$$

The CBAM attention mechanism can be utilized in UAV images, where there is a large and intensive variation in the object scales. The detection performance of the CBAM could be improved through extracting the attention zone by eliminating noise and zeroing in on the essential items.

### 3.5. SPPF+

We proposed a modified version of the SPPF structure termed SPPF+, which leverages the concepts of feature reuse from the SPPF and the SPPCSPC. The SPPF+ component successively pools the feature maps using pooling kernels of varying sizes (13 × 13, 9 × 9, and 5 × 5) and a stride of 1. Different kernel sizes correspond to distinct receptive fields, and combining numerous characteristics facilitates the extraction of fine-grained object details from aerial photographs. In high-density scenes of aerial photographs, the interaction of feature information can be improved by extracting features from many receptive fields on a single feature map, leading to a more precise object location. An image may learn features at multiple sizes before fusing the local and global elements using maximum pooling and jump connections at many scales to increase the feature map’s representational depth. Max pooling is one such method, which takes a whole picture and splits it into many rectangular areas, with the maximum value from each zone being produced as a result. The max pooling procedure helps reduce redundancy, but often results in the disappearance of valuable features.

**Figure 6:**
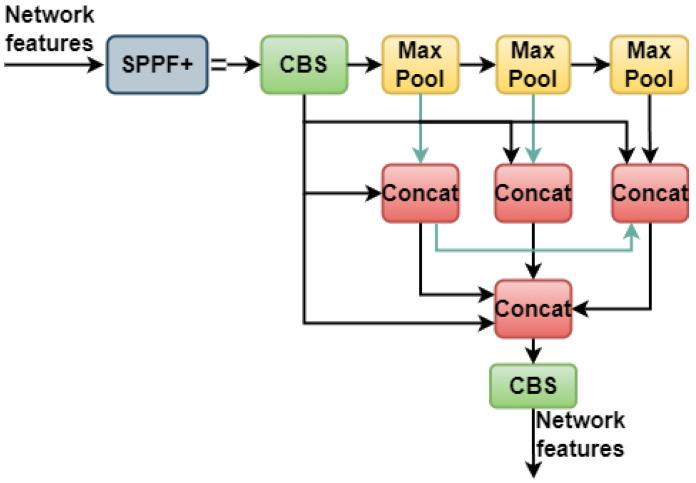
The structure of the SPPF+ module.

In this work, we strengthened the SPPF by developing dense linkages and promoting the concept of feature reuse. The SPPF module was then obtained, and the feature information loss associated with max pooling was mitigated. For the improved long-term recall of global information on small-target forest fires, the SPPF+ module was subsequently put to use. The SPPF+ module effectively retains global information and is useful for detecting small targets in forest areas affected by fires. A visual representation of the SPPF+ structure is shown in Figure 6.

### 3.6. BiFPN

Insufficient original data for learning can result in training deviations and negatively impact object detection accuracy. To address this issue, we improved the YOLO-V7 head portion using an enhanced bidirectional feature pyramid network (BiFPN) [25]. In the initial BiFPN, features from the feature extraction network were merged directly with the relative size features in the bottom-up path. A bidirectional channel was built using cross-scale connections and an additional edge. This allowed the network to maintain a sufficient deep semantic knowledge while preserving relatively superficial levels. To further improve feature fusion, the original BiFPN provided various input features with varied weights and used the same underlying structure several times.

**Figure 7:**
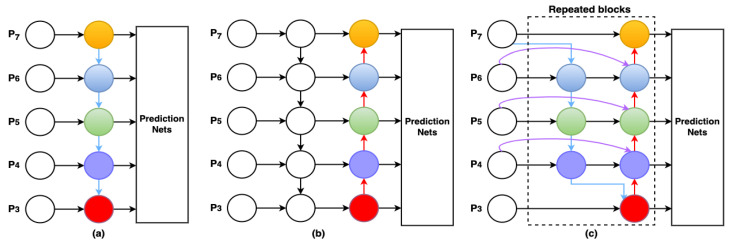
Differences between the FPN, PANet, and Bi-FPN designs. (**a**) In FPN, a top-down path is presented; (**b**) in PANet, an additional bottom-up path is added on top of the FPN; and (**c**) in Bi-FPN, each top-down and bottom-up path is treated as a separate layer in the feature network, with the same layer repeated several times to permit a more complicated feature fusion.

Multiscale feature fusion aims to combine information collected at several spatial and temporal resolutions [65]. For example, when given a set of features at various scales termed as $P^{in}=(P_{l_{1}}^{in},P_{l_{2}}^{in},\ldots )$, where $P_{l_{i}}^{in}$ represents the feature at level $l_{i}$, we want to discover a mapping function *f* that can efficiently merge these characteristics into a new set of features in the form of $P^{out}=f(P^{in})$. The top-down FPN [66] shown in Figure 7a is representative of the norm. Pin consists of elements from levels 3–7 of the input picture, represented by $P^{in}=(P_{3}^{in},\ldots P_{7}^{in})$, where $P_{i}^{in}$ is the feature level from the input image, and *½* is its size. If the input size is 640 × 640 pixels, for example, $P_{3}^{in}$ corresponds to feature level 3 (640/$2^{3}$ = 80) with a resolution of 80 × 80 pixels, while $P_{7}^{in}$ corresponds to feature level 7 with a size of 5 × 5 pixels, respectively. The conventional FPN performs a top-down merging of the multiscale characteristics as follows:

(3) $$P_{7}^{out}=Conv\left(P_{7}^{in}\right),$$

(4) $$P_{6}^{out}=Conv\left(P_{6}^{in}+Resize\left(P_{7}^{out}\right)\right),$$

…

(5) $$P_{3}^{out}=Conv\left(P_{3}^{in}+Resize\left(P_{4}^{out}\right)\right).$$

In the context of multiscale feature fusion, the operation *Resize* has been commonly used to adjust the size of feature maps to match each other, and *Conv* refers to a convolutional operation that is applied to extract features from the feature maps.

The traditional FPN’s multiscale feature fusion is limited by its top-down data flow. To address this, PANet adds a bottom-up path assembly network, as shown in Figure 7b. Bi-FPN has often been used with level 3–7 features obtained from the backbone network to perform bidirectional feature fusion. This merged information set can then be used to predict the object’s class and bounding box by the box and class networks. All feature levels contribute equally to the weights used by the box and class networks. Bi-FPN improves YOLOv7 by allowing for both top-down and bottom-up multiscale feature fusions with the use of learnable weights, thereby making the process both more convenient and faster. Regarding real-time wildfire smoke detection, Bi-FPN outperforms PANet, since it requires fewer parameters and FLOPS without sacrificing its accuracy.

### 3.7. Decoupled Head

**Figure 8:**
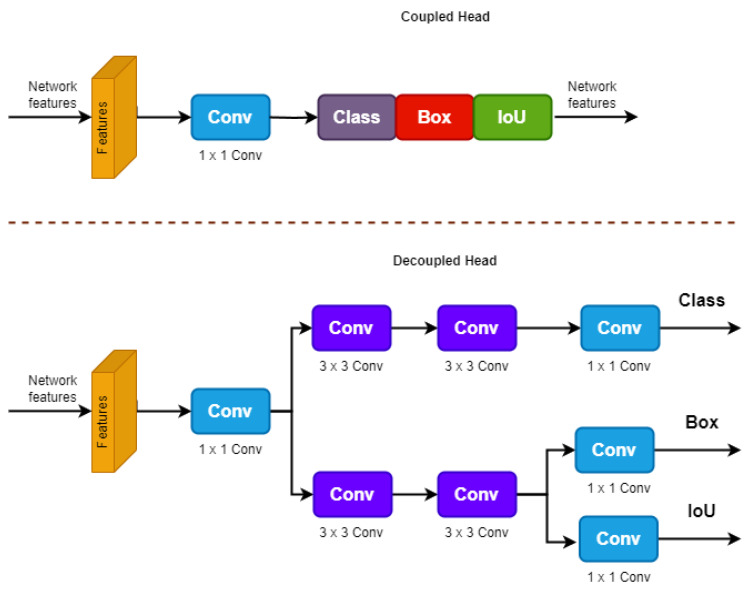
The architecture of the decoupled head and the coupled head.

The decoupled head in YOLOx [67] separates the classification and localization operations, which helps improve the detection accuracy. There are more convolutional layers for prediction, regression, and classification in the YOLOx decoupled head compared to the YOLOv7 coupled head. For each level of feature, the decoupled head includes a 1 × 1 convolutional layer for channel dimension reduction, followed by two parallel branches consisting of two 3 × 3 convolutional layers. In both branches, there is an additional 1 × 1 convolutional layer. The regression branch also includes an IoU branch. As shown in Figure 8, the decoupled head has more parameters, but it improves the converging speed as a result.

## 4. Experimental Results and Analysis

The experimental setup, test dataset, hyperparameters, and validation of the effectiveness of the enhanced YOLOv7 in detecting forest fire smoke in UAV pictures are all described in this section. All experiments were run on identical hardware to ensure the suggested approach’s reliability. All tests were performed on a custom-built personal computer with features such as Nvidia GeForce 1080Ti graphics processing units, a 32 GB of random access memory, and a nine core, 4.90 GHz central processing unit [68], as shown in Table 3. The input pictures for the improved YOLOv7 model were 640 × 640 pixels, obtained from a forest fire smoke dataset. The comprehensive evaluation includes a wide range of factors, such as the experimental setup and design, YOLOv7 performance analysis, method impacts analysis, model comparisons, ablation study, and visualization outputs.

### 4.1. Evaluation Metrics

In this work, we conducted quantitative tests using the widely used Microsoft COCO benchmarks (in Table 4) to assess the efficacy of the suggested approach, in line with the prior studies [18,68,69,70,71]. One way to measure a classifier’s accuracy is to count the times it correctly categorizes a given object. On the other hand, the recall of a model is the proportion of its correct predictions to the total quantity of ground truths. It is another indicator of its capacity to indicate critical situations correctly. When a model has a high recall, it may accurately identify a large proportion of ground-truth items while still retaining a high level of precision by focusing on only identifying the relevant objects. An ideal model would have a false-negative rate of zero, a recall rate of one, and an accuracy rate of one, respectively. By comparing the proposed system’s outputs with the ground-truth photos at the pixel level, and then calculating the precision and recall using suitable formulas, we were therefore able to assess the accuracy and recall rates of the proposed smoke detection method.

(6) $${Precision}_{C_{ij}}=\frac{{TP}_{C_{ij}}}{{TP}_{C_{ij}}+{FP}_{C_{ij}}},$$

(7) $${Recall}_{C_{ij}}=\frac{{TP}_{C_{ij}}}{{TP}_{C_{ij}}+{FN}_{C_{ij}}}.$$

The number of correctly identified smoke regions is represented by *TP*, while false positives resulting from the mis-identification of non-smoke regions as smoke are represented by *FP*, respectively. False negatives occur when actual smoke regions are incorrectly identified as non-smoke regions, and are represented by *FN*. The average precision (*AP*) was determined using Equation (8) based on these values:

(8) $${AP}_{C_{ij}}=\frac{1}{m}\sum_{j=1}^{m}{Precision}_{C_{ij}}.$$

The rate of detection can be quantified in frames per second (*FPS*), which in this study represents the mean detection rate in terms of pictures per second. The formula used to determine the frames per second is as follows:

(9) $$FPS=\frac{1}{t}$$

where *t* is the average time required to process each picture.

In addition, we also measured the complexity of the model using the number of floating point operations per second (FLOPS), which reflects the amount of computation in the model. 

### 4.2. Quantitative Comparisons

To assess the effectiveness of our proposed model, we performed rigorous quantitative evaluations using the standard Microsoft COCO benchmarks, including recall, precision, and AP, as previously calculated by Equations (6)–(8). Given the varying sizes and distances of smoke in our dataset, including both the large and small particles at different proximities, we systematically assessed and compared the performance of various one-stage object detectors, including several members of the YOLO series in order to identify the optimal model for detecting smoke under forest fire scenarios.

Our research focused on detecting forest fire smoke using deep learning models to limit the devastation of forest ecosystems and protect lives. After careful consideration of our dataset, we chose to utilize the YOLOv7 model due to its ability to quickly detect smoke of varying sizes and directions. Single-stage detectors were found to be more suitable for emergency situations and real-time implementation compared to the state-of-the-art multiple-stage object detectors. The proposed forest smoke detection model builds on YOLOv7 and achieves superior results in AP, AP_50_, AP_75_, AP_S_, AP_M_, and AP_L_, respectively, compared to other object detectors. 

To fully assess the merits of the suggested approach, we compared it to other multi-stage object identification techniques, including Libra-R-CNN [72], Faster R-CNN [73], Cascade R-CNN [74], Mask R-CNN [75], CoupleNet [76], MegDet [77], and DeNet [78], as well as several single-stage object detection methods, including M2Det [79], RFBNet [80], FSAF [81], SSD [32], RefineDet [82], NAS-FPN [83], DeepSmoke [84], RetinaNet [85], EfficientDet [65], YOLOv3 [29], YOLOv4 [86], YOLOv5 [87], and YOLOv7, respectively [61]. Table 5 provides an in-depth evaluation of the wildfire smoke dataset’s performance with the improved YOLOv7 model and the multi-stage object detectors. When comparing the efficacy of various object identification models, we maintained consistency by always employing the same collection of training and testing photos of the smoke from the custom wildfire smoke dataset. It is also shown how the improved YOLOv7 model compares against other single-stage object detectors on the same dataset in Table 6. When comparing our proposed model to other object detectors, it excels in detecting forest fire smoke.

### 4.3. Qualitative Evaluation

**Figure 9:**
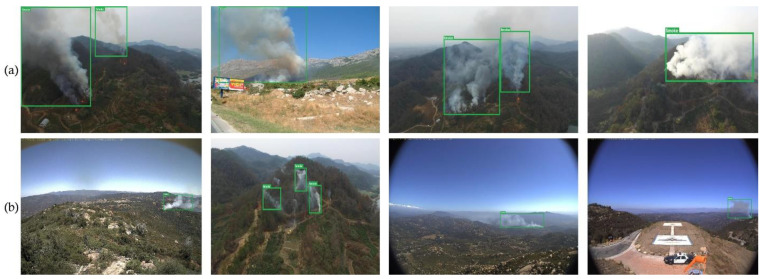
Sample results of smoke detection from forest fire: (**a**) massive plumes of smoke, and (**b**) minor plumes of smoke.

In addition to a quantitative evaluation of the suggested approach for detecting smoke from wildfires, we also conducted a qualitative study. To achieve this, we chose eight pictures from our dataset, four of which showed massive plumes of smoke from a forest fire and the four showing minor plumes of smoke that arose spontaneously. Using the optimized YOLOv7 model, we were able to obtain consistent and reliable results for both categories, as shown in Figure 9. These images depicted diverse scenes and conditions, including smoke traveling in different directions.

According to the literature, several strategies for identifying smoke from minor wildfires in photos have so far failed. We collected photographs of forest fire smoke in various sizes in an effort to enlarge the dataset and improve the accuracy of smoke detection. Figure 9b shows smoke pictures that are on the smaller side. To identify small-moving objects while preserving the detailed features, we adopted an approach inspired by [18] that combines a feature map from a preceding layer with a large-scale feature map. This extensive feature map can recognize smoke pixels of varied sizes by combining the location data from lower levels with the complicated properties from the upper layers.

Figure 9 shows that the suggested approach for identifying wildfire smoke, which employed the enhanced YOLOv7 model, can detect smoke in a wide variety of forest scenarios. The stability of the proposed technique was assessed using both big and small smoke pictures in the trials. To prevent and put out forest fire, early smoke detection is essential. If allowed to spread, even a tiny quantity of smoke can start a devastating wildfire that threatens human lives, forest resources, and the environment. The suggested approach can also identify small patches of smoke in photos with a high accuracy.

Our results show that the suggested technique can efficiently eliminate false detections, thereby allowing for early suppression and quick response times under all scenarios when forest fire smoke is present, despite its direction, scope, or form. Small amounts of smoke with a similar color and intensity levels as the surrounding scenery are often misidentified as smoke by the conventional visual fire detectors.

### 4.4. Ablation Experiments

Firstly, to conduct ablation investigations to assess the usefulness of the various attention mechanisms, we replaced the CBAM modules with the SE and ECA modules. The SE attention mechanism was designed to capture the relationship between global and local information in a flexible manner. In doing so, the model can identify an object’s important areas and assign them more weight, thereby emphasizing the relevant features and suppressing the irrelevant ones, leading to an improvement in the accuracy. The ECA module presents an alternative method of cross-channel interaction that does not rely on dimensionality reduction. This tactic protects the learning effect of channel attention from the detriment of dimensionality reduction. The ECA module can gather local cross-channel interaction data by evaluating the current channel and its k nearest neighbors. This little building component exhibits a significant effect despite having few moving parts.

To validate the efficacy of the enhanced algorithm, the proposed study incorporated the CBAM as the attention mechanism in the YOLOv7 model and performed investigations on the custom smoke dataset. Table 7 displays the results obtained using the AP, AP_50_, AP_75_, AP_S_, AP_M_, and AP_L_ metrics of the assessment.

Table 7 illustrates the comparison results of the ablation experiments using the improved YOLOv7 model and the SE and ECA modules added to the YOLOv7 model. Compared to the original YOLOv7 algorithm, the SE and ECA algorithms resulted in a lower accuracy, decreased recall, and reduced AP scores while increasing the model computational pressure parameters. In contrast, the CBAM attention mechanism performed better with increased average precision scores. The CBAM, which included spatial and channel attention mechanisms, was found to have outperformed the SE and ECA modules.

Secondly, the current study included ablation experiments to evaluate whether the SPPF+, BiFPN, and decoupled head (DP) modules enhance the accuracy of the proposed YOLOv7 smoke detection model. A total of eight ablation experiments were performed, including YOLOv7, YOLOv7 + (SPPF+), YOLOv7 + BiFPN, YOLOv7 + DP, YOLOv7 + (SPPF+) + BiFPN, YOLOv7 + (SPPF+) + DP, YOLOv7 + BiFPN+ DP, and YOLOv7 + (SPPF+) + BiFPN + DP, respectively. Experiments 2–8 were run in order, with the first two involving training the original YOLOv7 model with the addition of just SPPF+, the second two involving training the original YOLOv7 model with the addition of only BiFPN, and the third and final experiment including training the original YOLOv7 model with the acquisition of the DP, respectively. The results of the ablation experiments are shown in Table 8, and they suggest that the proposed changes can improve the YOLOv7 model’s performance.

Ablation research has shown that although YOLOv7 is a popular object detection model, it produces unsatisfactory results. These results indicate that upgrading the network topology in YOLOv7 with SPPF+, BiFPN, and DP may significantly enhance the model’s accuracy.

## 5. Discussion

Unlike other vision inspection tasks, including face identification, defect detection, and lane line detection, detecting forest fire smoke has its distinct obstacles. The identification task is made more difficult by the ever-shifting smoke target’s irregular form, and by the presence of many interfering variables in the complicated woodland environment, such as haze and clouds. A minor fire might quickly become a large-scale tragedy with catastrophic damages if its discovery is delayed or overlooked. Using computer vision technology to replace human inspection is an effective way to deal with these issues due to its many benefits. Computer vision enhances smoke detection, allowing for the early and exact identification of possible fire breakouts. As a result, reaction times are reduced, and fire prevention and control are improved overall.

To multi-directionally detect smoke from forest fires, we curated a large dataset that includes many types of smoke properties. Multiple experimentation sets were compared and analyzed to verify the effectiveness of the proposed improved YOLOv7 model for forest fire smoke detection. The model successfully accounted for the existence of tilted smoke, the detection of small in size smoke, and the difficulty of distinguishing smoke from clouds and fog. Despite the abundance of the publicly available smoke image datasets, smoke detection research has historically been limited by a lack of dataset variety. Therefore, aerial pictures of forest fire smoke collected by UAVs at varied distances during fire occurrences formed the basis of a carefully curated object detection dataset.

Our model initially integrated the CBAM into the YOLOv7 network’s core infrastructure. To further concentrate smoke from localized wildfires, the SPPF layer of the backbone was updated to SPPF+. To further fine-tune the neck and accomplish a more exact fusion of characteristics across several scales, a BiFPN module was introduced as a third stage. Finally, decoupled heads were incorporated into YOLOv7 instead of coupled heads to further enhance detection results. These three improvements boosted the detection performance by 3.8–5.3% in terms of AP, AP50, AP75, APS, APM, and APL, respectively. The model’s detection capacity was enhanced across various scenarios thanks to the considerable increases in the average precision for small, medium, and large smoke.

However, despite its successes, the suggested forest fire smoke detection method has particular restrictions. Its sensitivity to atmospheric phenomena, including fog, haze, and clouds is a significant limitation, which might give the impression of smoke presence. Furthermore, having pixel values similar to those of a smoke plume is a substantial obstacle in cloudy or hazy environments. To improve the correct detection to locate the origin of forest smoke, we plan to invest in a technology that can identify the difference between the different-sized clouds and types of smoke. These enhancements were made to improve the model’s smoke prediction performance by increasing the quantity of the training data and extracting more valuable features from the data. One potential expansion of this area could be to use the size and form determination modules for smoke. In addition, our analysis was limited to daylight times. Therefore, we will concentrate on the model’s ability to spot wildfires at night in the future. Based on our research, smoke detectors may not be as effective as fire alarms under dark environments.

Our future efforts will address the model’s tendency to produce many false positives under challenging conditions, such as under low-altitude cloud cover and haze. Since fires tend to occur in the same places and under the same conditions during particular months, we want to improve our predictions by including additional information, such as fire location, date, and previous meteorological data. The incompatibility of the suggested method with edge devices is another area for enhancement. However, this problem could be fixed by decreasing the model size without sacrificing its performance. By using distillation techniques to train a smaller deep network, such as YOLOv7-tiny, we present the possibility of constructing a model tailored for edge computing while retaining the same level of performance as our existing model.

## 6. Conclusions

Forest fire smoke detection algorithms need better performance since collecting enough training images is difficult, leading to problems, including data imbalance and overfitting. This research provides an optimized YOLOv7 model for identifying smoke from forest fires in complicated wildland scenes. It can be seen from Table 7 that these improvements, such as SPPF+, BiFPN, and decoupled heads achieved an AP of 78.6%, AP_50_, of 86.4%, and AP_75_ of 81.7, respectively, which improved AP, AP_50_, and AP_75_ by 2.7%, 3%, and 5.5%, respectively. In terms of the ablation study on attention mechanism, the CBAM was found to perform consistently better with an AP_50_ of 83.4%, compared to ECA and SE with AP_50_s of 83.2% and 82.9%, respectively. Experimental results revealed that the optimized YOLOv7 model outperformed the state-of-the-art and multiple-stage object detection models on the custom smoke image dataset, with an AP_50_ of 86.4% and an AP_L_ of 91.5%, respectively. Furthermore, while YOLOv7 showed the second-best result for AP_50_ and an AP_L_ with 82.5% and 87.3%, respectively, the traditional forest fire smoke detection sensor was confined to a small, contained area, and can only detect one fire at a time. This new and enhanced YOLOv7 technology eliminates these problems. Outdoor smoke detection with temporal and geographical features is possible under the current conditions. This research presents a novel model for a high-performance detection network that could be used to spot the smoke of forest fires.

Improving the quality of smoke photos is essential for advancing smoke detection in wildland scenarios. Therefore, future research will focus on gathering a wide and diverse collection of forest fire smoke datasets and using image enhancement techniques. Compressing the model is another avenue we will explore to speed up detection without sacrificing precision.

## Figures and Tables

**Table 1:** Forest fire smoke dataset and its specification.

Dataset	Smoke Images				Non-Smoke Images				Total
	Google	Kaggle	Flickr	Bing	Google	Kaggle	Flickr	Bing	
Forest smoke	350	2600	200	350	150	2550	100	200	6500

**Table 2:** Data augmentation on the forest fire smoke dataset.

Forest Smoke	Training Images			Testing Images	Total
	Original Images	Rotated Images	Flipped Images	Original Images	
Smoke images	2800	5600	8400	700	17,500
Non-smoke images	2400	4800	7200	600	15,000
Total	5200	10,400	15,600	1300	32,500

**Table 3:** Specification of the hardware and software.

Components	Specifications	Descriptions
GPU	GPU 2-GeForce 1080	Two GPU’s were installed
CPU	Intel Core 9 Gen i7-9700k (4.90 GHz)	
RAM	DDR4 32 GB (DDR4 16 GB × 2)	Samsung DDR4 16 GB PC4-21300
Storage	SSD: 512 GB/HDD: TB (2 TB × 2)	
Motherboard	ASUS PRIME Z390-A STCOM	
OS	Ubuntu desktop	Version: 18.0.4 LTS

**Table 4:** The precision and recall of object detection models, which are commonly evaluated using Microsoft’s COCO benchmarks at various levels.

AP at different levels	AP_50_	AP at IoU = 0.5
	AP_75_	AP at IoU = 0.75
	AP_S_	AP_0.5_ for small area: area < $32^{2}$
	AP_M_	AP_0.5_ for medium area: $32^{2}<$ area < $96^{2}$
	AP_L_	AP_0.5_ for large area: area > $96^{2}$

**Table 5:** Comparison results between the proposed method and multiple-stage object detectors.

Model	AP	AP_50_	AP_75_	AP_S_	AP_M_	AP_L_	FPS
Mask R-CNN [75]	68.6	76.9	72.1	60.5	67.5	80.2	-
Fast R-CNN [73]	62.7	69.5	63.6	53.1	61.5	74.3	-
Faster R-CNN [33]	64.9	71.8	66.5	55.7	63.5	75.4	-
Cascade R-CNN [74]	71.4	79.6	75.4	63.9	70.5	84.8	-
Libra-R-CNN [72]	53.5	64.6	60.4	45.2	52.8	69.5	-
CoupleNet [76]	59.7	66.5	61.8	50.4	59.2	71.7	-
DeNet [78]	56.3	65.4	59.7	47.3	57.5	71.6	-
MegDet [77]	63.4	72.3	66.5	54.8	62.7	77.2	-
The proposed	78.6	86.4	81.7	70.3	77.2	91.5	153

**Table 6:** Comparison results between the proposed method and single-stage object detectors.

Model	AP	AP_50_	AP_75_	AP_S_	AP_M_	AP_L_	FPS
M2Det [79]	59.3	69.5	63.7	51.4	58.6	74.8	26.3
FSAF [81]	59.6	69.8	63.9	51.7	59.3	75.2	22.7
RFBNet [80]	63.5	69.4	64.3	52.5	60.2	73.9	25
EfficientDet [65]	71.8	78.3	74.6	63.7	70.4	83.8	28.5
NAS-FPN [83]	62.4	72.1	66.5	54.3	61.8	76.3	20.4
SSD [32]	64.5	72.6	66.3	55.8	64.7	77.2	82.6
RefineDet [82]	69.2	76.5	71.8	60.9	67.6	82.4	61.5
DeepSmoke [84]	72.3	79.7	75.4	64.6	71.5	86.2	35.4
RetinaNet [85]	66.4	73.8	68.2	57.6	64.3	69.7	67.2
YOLOv3 [29]	68.6	76.3	69.5	60.1	67.8	79.6	31.8
YOLOv4 [86]	70.7	78.5	72.6	61.4	69.2	82.8	35.6
YOLOv5 [87]	71.9	79.2	73.4	63.8	70.5	84.5	156
YOLOv7 [61]	74.8	82.5	75.4	67.2	73.6	87.3	161
The proposed	78.6	86.4	81.7	70.3	77.2	91.5	153

**Table 7:** Comparison results of ablation study for attention mechanisms.

Model	Attention Mechanism			Evaluation Metrics								
	CBAM	ECA	SE	AP	AP_50_	AP_75_	AP_S_	AP_M_	AP_L_	FPS	GFLOPS	Latency
YOLOv7	×	×	×	74.8	82.5	75.4	67.2	73.6	87.3	161	105.7	12 ms
	√	×	×	75.9	83.4	76.2	68.1	74.5	88.3	157	105.5	8 ms
	×	√	×	75.5	83.2	75.8	67.8	74.2	88	152	105.3	9 ms
	×	×	√	75.3	82.9	75.7	67.6	73.8	87.7	158	105.8	11 ms

**Table 8:** Comparison results of the ablation study for various modules.

Model	Modules			Evaluation Metrics								
	SPPPF+	BiFPN	DP	AP	AP_50_	AP_75_	AP_S_	AP_M_	AP_L_	FPS	GFLOPS	Latency
YOLOv7 + CBAM	×	×	×	75.9	83.4	76.2	68.1	74.5	88.3	157	105.9	8 ms
	√	×	×	76.7	84.3	78	68.7	75.3	89.2	162	106.2	12 ms
	×	√	×	76.9	84.5	78.2	69	75.5	89.5	156	104.8	9 ms
	×	×	√	76.8	84.4	77.9	68.8	75.4	89.4	154	104.5	8 ms
	√	√	×	77.7	85.4	80	69.6	76.3	90.4	158	106	11 ms
	×	√	√	77.8	85.5	79.9	69.7	76.4	90.6	155	104.6	8 ms
	√	×	√	77.6	85.3	79.7	69.4	76.2	90.3	160	106.1	10 ms
	√	√	√	78.6	86.4	81.7	70.3	77.2	91.5	153	104.2	7 ms

## Data Availability

Not Applicable.

## Abbreviations

ABi-LSTM	Attention enhanced bidirectional long short-Term memory network
AI	Artificial intelligence
AP	Average precision
BiFPN	Bidirectional feature pyramid network
CAM	Channel attention module
CBAM	Convolutional block attention module
CBS	Convolution, batch normalization, SiLU activation function
CIoU	Complete intersection over union
CNN	Convolutional neural network
CSP	Convolutional spatial pyramid
DOTA	Dataset for object detection in aerial images
DP	Decoupled head
ECA	Efficient channel attention
E-ELAN	Extended efficient layer aggregation network
FLAME	Fire luminosity airborne-based machine learning evaluation
FLOPS	Floating point operations per second
FPN	Feature pyramid network
FP	False positive
FPS	Frame per second
FN	False negative
GAP	Global average pooling
GB	Gigabyte
GPU	Graphics processing unit
GFLOPS	GPU floating point operations per second
GHz	Gigahertz
GMP	Global max pooling
GIoU	Generalized intersection over union
GUI	Graphical user interface
IoU	Intersection over union
Microsoft COCO	Common objects in context
NEMO	Nevada smoke detection benchmark
PANet	Path aggregation network
ReLU	Rectified linear unit
SAM	Spatial attention module
SE	Squeeze-and-excitation
SiLU	Sigmoid linear unit
SPPCSPC	Spatial pyramid pooling cross stage partial network
SPPF+	Spatial pyramid pooling fast+
SSD	Single-shot detector
TN	True negative
TP	True positive
UAV	Unmanned aerial vehicle
VGG16	Visual geometry group 16
YOLO	You only look once
